# Prevalence of Pulmonary Hypertension in an Unselected Community-Based Population: A Retrospective Echocardiographic Study—RES-PH Study

**DOI:** 10.3390/jpm11060489

**Published:** 2021-05-31

**Authors:** Egidio Imbalzano, Marco Vatrano, Alberto Lo Gullo, Luana Orlando, Alberto Mazza, Vincenzo Antonio Ciconte, Vincenzo Russo, Clemente Giuffrida, Pierpaolo Di Micco, Antonio Giovanni Versace, Giuseppe Mandraffino, Giovanni Squadrito

**Affiliations:** 1 Department of Clinical and Experimental Medicine, University of Messina, 98100 Messina, Italy; luana_orlando@libero.it (L.O.); agversace@unime.it (A.G.V.); gmandraffino@unime.it (G.M.); giovanni.squadrito@unime.it (G.S.); 2 UTIC and Cardiology, Hospital “Pugliese-Ciaccio” of Catanzaro, 88100 Catanzaro, Italy; marco.vatrano1975@gmail.com (M.V.); enzocico2003@yahoo.it (V.A.C.); 3 Unit of Emergency Medicine, Irccs Neurolesi Bonino Pulejo, 98100 Messina, Italy; alberto.logullo@gmail.com (A.L.G.); clemente.giuffrida@irccsme.it (C.G.); 4 Internal Medicine Unit, Azienda ULSS 5 Polesana—Rovigo General Hospital, 45100 Rovigo, Italy; mazza.alberto@azisanrovigo.it; 5 Department of Medical Translational Sciences, Division of Cardiology, Monaldi Hospital, University of Campania “Luigi Vanvitelli”, 80100 Naples, Italy; vincenzo.russo@unicampania.it; 6 Department of Medicine, Buonconsiglio Fatebenefratelli Hospital, 80122 Naples, Italy; pdimicco@libero.it

**Keywords:** echocardiography, pulmonary hypertension, pulmonary artery systolic pressure, unselected population

## Abstract

Introduction. The actual prevalence of pulmonary hypertension (PH) in Italy is unknown. Echocardiography is useful in the screening of patients with suspected PH by estimation of the pulmonary artery systolic pressure (PASP) from the regurgitant tricuspid flow velocity evaluation, according to the simplified Bernoulli equation. Objectives. We aimed to evaluate the frequency of suspected PH among unselected patients. Methods. We conducted a retrospective cross-sectional database search of 7005 patients, who underwent echocardiography, to estimate the prevalence of PH, between January 2013 and December 2014. Medical and echocardiographic data were collected from a stratified etiological group of PH, using criteria of the European Society of Cardiology classifications. Results. The mean age of the study population was 57.1 ± 20.5 years, of which 55.3% were male. The prevalence of intermediate probability of PH was 8.6%, with nearly equal distribution between men and women (51.3 vs. 48.7%; *p* = 0.873). The prevalence of high probability of PH was 4.3%, with slightly but not significant higher prevalence in female patients (43.2 vs. 56.8%; *p* = 0.671). PH is predominant in patients with chronic obstructive pulmonary disease (COPD) or left ventricle (LV) systolic dysfunction and related with age. PASP was significantly linked with left atrial increase and left ventricular ejection fraction. In addition, an increased PASP was related to an enlargement of the right heart chamber. Conclusions. PH has a frequency of 4.3% in our unselected population, but the prevalence may be more relevant in specific subgroups. A larger epidemiological registry could be an adequate strategy to increase quality control and identify weak points in the evaluation and treatment of these patients.

## 1. Introduction

Pulmonary hypertension (PH) is a clinical syndrome characterized by breathlessness, fatigue, and weakness, with a gradual increase of pulmonary vascular resistance and ultimately right heart failure [1,2]. PH can be idiopathic, familial or related to different diseases, such as congenital heart disease, connective tissue disease, HIV infection, portal hypertension, and drugs [3]. Pulmonary vascular obstruction triggered by intima and media proliferation represents the key point of the pathogenesis of PH [4]. Vascular remodeling and thrombosis lead to an increase of pulmonary vascular resistance in pulmonary hypertension [5].

The gold standard of PH for diagnosis is right heart catheterization (RHC), which is not suitable or practical to perform in population studies. Doppler echocardiography is a non-invasive key screening tool in the diagnostic algorithm and prognostic assessment [6]. Pulmonary artery systolic pressure (PASP), computed by the regurgitant tricuspid flow velocity evaluation, according to the simplified Bernoulli equation, is the most used and largely accepted parameter [7].

There is little information on the prevalence of pulmonary hypertension in the general population, and population-based studies are really limited [8,9,10,11]. Therefore, we thought to characterize the prevalence of PH as evaluated by echocardiography in the unselected population in Southern Italy.

## 2. Patients and Methods

We conducted a retrospective study using an echocardiography database search of 7005 patients that were referred to our unit for transthoracic echocardiography, between January 2013 and December 2014. All the eligible patients were included in the registry, in the period indicated, and excluded all those who could not be recruited for history or clinical data. Patients gave previously an informed consent to be enrolled in clinical studies and, the Ethics Committee of Messina, University Hospital, reviewed and approved the study.

All echocardiograms were collected by experienced sonographers using a GE Vivid 7 or Vivid E9 (GE Vingmed Ultrasound AS, Horten, Norway) cardiac ultrasound machine. The protocol included a two-dimensional study in the parasternal axis as well as apical and subcostal views. PASP was calculated as the sum of the estimated right atrial pressure (RAP) by an inferior vena cava diameter and the peak velocity of the tricuspid regurgitant jet (TRV) as: PASP = 4 × TRV^2^ + RAP. For standardization, a RAP of 10 mmHg was assumed for all patients unless clear features were present that suggested otherwise. Patients were prospectively stratified into three diagnostic probability groups of pulmonary hypertension: Low probability with PASP ≤ 36 mmHg, intermediate probability with PASP between 37 and 50 mmHg, high probability with PASP > 50 mmHg. In addition, the patients were grouped by the presence of the right atrial (RA, area > 18 cm^2^) and ventricular (RV, diameter > 3.65 cm) enlargement, according to the guidelines of the American Society of Echocardiography [12,13].

PH etiology was evaluated using criteria and subcategories of the ESC classifications (group 1: Idiopathic PH; group 2: PH in association with left heart disease; group 3: PH related to hypoxic lung disease; group 4: PH due to chronic thrombo-embolic disease (CTEPH); and group 5: Miscellaneous) [1]. We considered as demographical data (age, sex, BMI, and smoking) and as clinical data (hypertension, coronary artery disease, arrhythmic heart disease, valvular disfunction, and chronic obstructive pulmonary disease). Moreover, we looked at concurrent medications such as statins, diuretics, ACE inhibitors, Beta-Blockers, and dual antiplatelet therapy (see Table 1). Clinical data and echocardiographic results were used to classify patients in different groups of PH as mentioned above. Where required, the patient’s general practitioner was consulted for more detailed information. In patients with two or more known causes of PH, the dominant cause was classified. This study fits with the STROBE recommendations for reporting cross-sectional studies [14].

## 3. Statistical Analysis

The test for normality was carried out on all variables by the Kolmogorov-Smirnov test. Since the main study variables presented a normal distribution, also considering the wide sample size, a parametric approach was chosen. Consistently, data are presented as the mean ± standard deviation and frequency of occurrence (%) where appropriate. ANOVA with the post-hoc Tukey test was performed to verify any difference among the groups and the between-groups difference was tested by the paired Student’s test. The interdependence analysis was carried out by Pearson’s correlation test. The missing data were imputed with a multiple imputation procedure (five imputations) using the Markov Chain Monte Carlo method.

The intra- and inter-observer agreement (two different observers, two readings for each observer) for the PASP evaluation and Simpson’s method were assessed by linear regression with the Bland-Altman analysis, showing a correlation of 0.91 and 0.88, respectively. To identify significant independent determinants of PASP, we performed a multivariate linear regression analysis including relevant clinical data (age, sex, body mass index, blood pressure, and heart rate) and standard echocardiographic measurements (left and right ventricular and atrial dilatation encoded as dichotomic variables, left ventricular mass index, left ventricular ejection fraction, Doppler transmitral inflow values, and TAPSE). Then, we performed an additional multivariate analysis in the group with possible and likely PH. A *p*-value < 0.05 was considered statistically significant. The SPSS 20 statistical software was used for the analysis (Statistical Package for Social Sciences, Chicago, IL, USA).

## 4. Results

**Figure 1:**
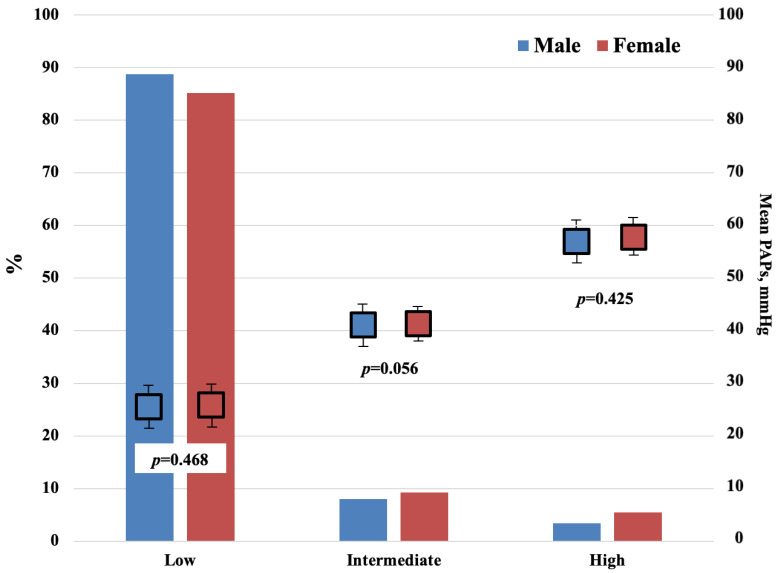
Histogram distribution of population as a function of PH probability, mean PASP, and gender. In addition, 5674 patients with PASP ≤ 36 mmHg are classified as low probability of PH (87.1%), 559 patients with PASP between 37–50 mmHg are classified as intermediate probability of PH (8.6%), 280 individuals with PASP > 50 mmHg were diagnosed as high probability of PH (4.3%). No significant difference was found between the men and women in each group.

Between January 2013 and December 2014, 7005 echocardiography studies were performed in 7005 patients for various reasons, including investigations of murmurs, ventricular function, breathlessness, and pulmonary hypertension. In addition, 492 patients were excluded, due to missing echocardiographic recording data, especially the PH value or clinical, anamnestic or instrumental data that did not allow the etiology of PH. Therefore, the ESC classification criteria were used. For this study, 6513 patients were qualified (patients’ mean age 57.1 ± 20.5 years, male 55.3%), clinical and demographic characteristics are shown in Table 1. Of these qualified patients, 5674 had PASP ≤ 36 mmHg and therefore were classified as low probability individuals with PH (87.1%), 559 had echocardiography evidence of PASP between 37 and 50 mmHg with a diagnosis of intermediate probability of PH (8.6%), and 280 individuals with PASP > 50 mmHg were diagnosed as high probability patients with PH (4.3%). Figure 1 shows the frequencies of low, intermediate, and high probability of PH and the mean PAPs values, according to sex without gender differences (low M/F, *p* = 0.468; intermediate M/F, *p* = 0.056; high M/F, *p* = 0.425). Of the 839 patients with elevated PASP (both intermediate and high probability group of PH), 675 patients (80.5%) had left heart disease as the dominant cause, 85 (10.1%) respiratory disease, 14 (1.7%) chronic pulmonary thromboembolic disease, and 43 (5.1%) other diseases (“miscellaneous”) causing PH. Seven patients (0.8%) had congenital heart disease-associated PH, and 15 patients (1.8%) had connective tissue disease-associated PH.

**Figure 2:**
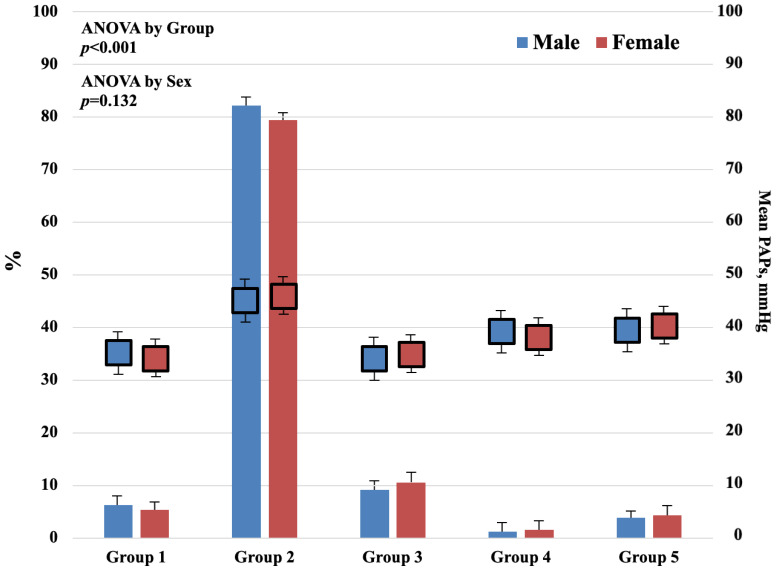
Mean PASP in the study population, classified into five groups, using criteria of the ESC classification. Group 1: Idiopathic PH; group 2: PH in association with left heart disease; group 3: PH related to hypoxic lung disease; group 4: PH due to chronic thrombo-embolic disease (CTEPH); group 5: Miscellaneous. The second group is the most common in our study. A significant difference of mean PASP by group (*p* < 0.001) is calculated. No significant difference between men and women (*p* = 0.132).

As shown in Figure 2, the second group is the most common in our study, with a significant difference in mean PAPs by group (*p* < 0.001) and nearly equal distribution between men and women (*p* = 0.132).

Among the patients with higher values of PASP, females with comorbidities (*p* = 0.001) and evidence of systolic and diastolic left and/or right ventricular dysfunction (both *p* = 0.001) were more prevalent. Data are shown in Table 2.

The prevalence of intermediate probability of PH was 8.6%, with nearly equal distribution between men and women (51.3 vs. 48.7%; *p* = 0.873). The prevalence of high probability of PH was 4.3%, with slightly but not significant higher prevalence in female patients (43.2 vs. 56.8%; *p* = 0.671). PH is more relevant in patients with chronic obstructive pulmonary disease (COPD) or left ventricle (LV) systolic dysfunction.

**Figure 3:**
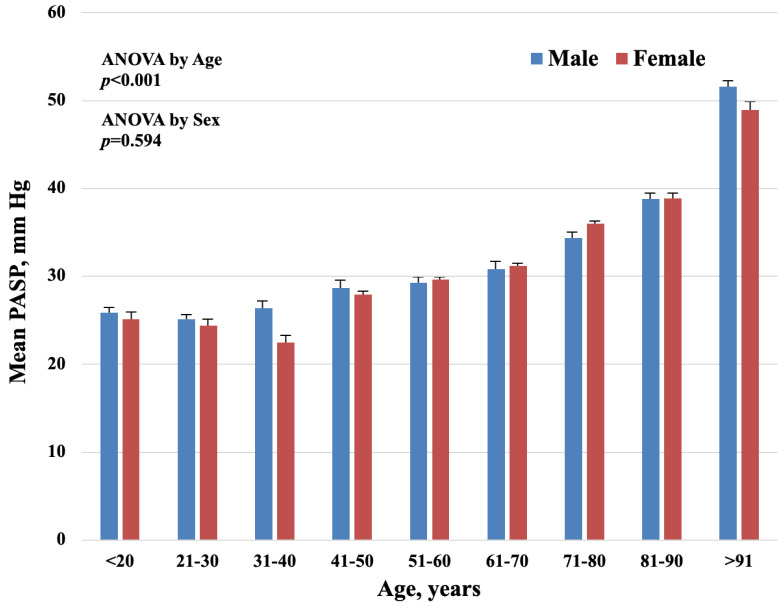
Distribution of study population by age shows a significative increase in age-related mean PASP (*p* < 0.0001). No significant difference by sex (*p* = 0.594).

PASP values increased significantly with age (*p* < 0.0001) without gender differences. Data are shown in Figure 3.

The interdependence analysis showed a significant correlation between PASP and left atrium size (*r* = 0.354, *p* < 0.0001) and left ventricular ejection fraction (*r* = −0.235; *p* < 0.0001). In addition, PASP was also correlated to the right atrial size (*r* = 0.272, *p* < 0.0001) and right ventricle size (*r* = 0.221, *p* < 0.0001).

By the multivariate logistic regression analysis in the overall study population, age, female sex, right atrium/right ventricle/left atrial dilatation, left ventricular ejection fraction (LVEF), and tricuspid annular plane systolic excursion (TAPSE) were independent predictors of PASP, as shown in Table 3.

In the following multivariate analysis performed in patients with intermediate and high probability of PH, only female sex and the right ventricular dilatation remained independent predictors of elevated values of PASP.

## 5. Discussion

Our study, performed on a heterogeneous population in Southern Italy, represents the first epidemiological evidence on the distribution of various categories (groups) of PH, in a reference transthoracic echocardiography laboratory. Although PH is burdened by increased mortality and early diagnosis is required, data on the prevalence of increased PASP in the general population is poor. Our indicative prevalence of PH was 12.9% with nearly equal distribution between men and women, while more specifically, a high probability PH was only 4.3%. These results are consistent with data reported in healthy volunteers from Italy, where PASP > 40 mmHg was recently estimated at 8% [15], while it was reported to be 6.6% in the INCIPIT study [16]. The same results were reported in an African-American population with a PH prevalence of 6.8% [17], while the authors of the Armadale study reported a prevalence of 9.1% [8]. Instead, in the Rotterdam study, the frequency of PH was 2.6% used as a cut-off value of PAPS > 40 mmHg or a right ventricular end-diastolic dimension > 42 mm: The prevalence increased up to 4.5% especially in the male sex (6.2 vs. 3.2%, *p* < 0.001) and in older participants [11]. In the French registry, the prevalence of isolated PH was 15 cases/million [10], while in the Scottish registry PH was 52 cases per million [18]. The REVEAL registry that described lower estimates of PH prevalence was classified as group 1 (12.4/million) [9].

We have shown an increased prevalence of age-related PASP, regardless of other diseases and cardiopulmonary function. These data are consistent with the observations, confirming the increase of PASP pressures with the increasing age in the REVEAL [9]. McQuillan et al. showed that mean echocardiography PASP values were 28 ± 5 mmHg in 3790 normal subjects [19] and increased with age, as shown in the present report. Moreover, in a study from the Olmsted county cohort, it has been reported that pulmonary artery pressures increase with age in subjects from the general community [20]. In the Rotterdam study, the prevalence of PH is lower in younger patients matched to older participants [11]. The higher prevalence of PH in older people may be related to age-associated blood vessel stiffening as also occurs in the systemic arteries [20]. Furthermore, we found that women have an increased value of PASP as compared to men. A role for hormones, such as oestrogens, may be suggested in the pathogenesis of PH [21]. Moreover, we observed an increased prevalence of PH in COPD patients according to pre-existent epidemiological evidence that identify COPD as the second most usual reason of PH, after left sided heart disease. The PH incidence is related to the severity of COPD, appearing in about half of patients with severe chronic obstructive pulmonary disease [22], in which prognosis is reduced with increased exacerbations of disease [23]. However, pulmonary vascular changes can anticipate the development of pulmonary hypertension in mild COPD [24,25]. In our cohort, the PH concomitant to respiratory disease, including COPD, showed the second most frequency: 9.5% of 559 patients with intermediate probability of PH had received a diagnosis of COPD, as well as the 11.1% out of the group with high probability of PH. This data is in line with the results from the Armadale cohort in which 9% had elevated PASP due to respiratory disease [8]. On the other hand, the study from the Rotterdam project found a lower prevalence (5.9%) compared to our data, probably due to the exclusion of severe cases of COPD [11].

In our cohort, the most common PH was group II (Figure 2), and higher values of PASP were found also in those patients with systolic and diastolic and left and right ventricular dysfunction. In group II, the pathophysiology mechanism of PH is a high pulmonary capillary wedge pressure due to the LV diastolic or systolic dysfunction with or without left-sided valvular disease [26]. In early-stage PH, the right ventricle fits with an afterload by increasing contractility with only slightly chamber enlargement [27]. However, when the right ventricular–arterial coupling is less than optimal, a reduction of aerobic exercise capacity can occur by limiting the maximum cardiac output [26,28]. In advanced stages, myocardial fibrosis, sarcomeric stiffening, and imbalance between the RV systolic function and afterload gradually lead to dilatation of the right heart chamber. These pathophysiological changes result in right ventricular increased pressures and consequent reduction in the filling pressure of the left ventricle, which, in turn, begins reducing the right flow and, eventually, the systemic arterial pressure and the left ventricular function, with exertional dyspnea and systemic venous congestion typical of PH [29,30]. In our study, a dilated right ventricle was the main predictor of increased PASP, whereas LV dilatation was not found to be independently associated with PASP, according to other studies without consistent relationships between ejection fraction and PASP [11,21].

The main strengths in our study are represented by the sample size. In addition, we could report the epidemiology of all the five groups of PH in real life. The main limitation is that we cannot validate the diagnosis of PH by right heart catheterization. However, invasive screening in a large simple size is not suitable and really challenging to perform. Transthoracic echocardiography is a valid instrumental examination in the general population with suspected PH, and becomes a very useful tool in patients with symptoms or risk factors, reaching high sensitivity (83%) and specificity (72%) [31]. Indeed, a recent statement by the Polish Cardiac Society Working Group confirmed the diagnostic usefulness of the echocardiogram on screening for high risk patients after acute pulmonary embolism [32].

In conclusion, echocardiographic PH estimation has low prevalence in our unselected population in Southern Italy, but the assessments may be higher in specific subgroups, especially in those with left ventricular dysfunction or COPD. Female sex and right ventricular dilatation were associated with PASP independently of heart or lung disease. The management of these patients in specialized centers guarantees a high quality of care. Based on the results of our epidemiological observation, we can confirm that registries are undoubtedly an essential tool in terms of quality control and could become necessary to identify weaknesses in the evaluation of patients with suspected pulmonary hypertension.

## Figures and Tables

**Table 1:** Clinical and demographic characteristics.

	All Patients (*n* = 6513 of 7005)
Male sex (%)	55.3
Age (year)	57.1 (20.5)
BMI (kg/m^2^)	28.1 (6.1)
Smokers (%)	60.6
HT (%)	5.6
CAD (%)	23.6
Valvular heart disease (%)	27.9
Arrhythmic disease (%)	3.6
COPD (%)	1.3
Total Cholesterol (mg/dL)	193.3 (44.0)
HDL Cholesterol (mg/dL)	42.3 (8.6)
Triglyceride (mg/dL)	134.2 (51.6)
Fasting glucose (mg/dL)	89.1 (13.9)
Creatinine (mg/dL)	0.9 (0.1)
Dual antiplatelet therapy (%)	54.2
Statins (%)	47.3
Diuretics (%)	27.7
ACE inhibitor (%)	63.3
Beta-blocker (%)	48.2
RAP (mmHg)	9.3 (3.4)
PASP (mmHg)	33.9 (11.8)
TAPSE (mm)	20.8 (4.5)
EF (%)	51.3 (11.4)
E/A ratio	0.8 (0.4)
WMSI	1.6 (0.3)

Baseline characteristics of enrolled population. BMI: Body mass index; HT: Hypertension; CAD: Coronary artery disease; COPD: Chronic obstructive pulmonary disease; HR: Heart rate; RAP: Right atrial pressure; PASP: Pulmonary artery systolic pressure; TAPSE: Tricuspid annular plane systolic excursion; EF: Ejection fraction; WMSI: Wall motion score index. Data are presented as the mean and standard deviation (SD) for variables with normal distribution, as the median and SD for variables with not-normal distribution, and as frequency (percent), where appropriate.

**Table 2:** Population distribution by PH probability.

	Low Probability < 36 mmHg (5674 pts)	Intermediate Probability 37–50 mmHg (559 pts)	High Probability 50 mmHg (280 pts)	*p*-Value
Male sex (%)	56.3	51.3	43.2	<0.001
Age (year)	55.2 (20.5)	72.4 (12.4)	74.2 (12.0)	<0.001
BMI (kg/m^2^)	27.9 (5.1)	27.6 (5.5)	28.8 (7.7)	0.431
Smokers (%)	58.3	62.3	61.2	0.563
HT (%)	6.2	1.6	1.1	<0.001
CAD (%)	22.5	30.6	31.4	<0.001
Valvular heart disease (%)	25.5	43.6	44.3	<0.001
Arrhythmic disease (%)	3.6	3.6	4.6	0.674
COPD (%)	0.4	9.5	11.1	<0.001
Total Cholesterol (mg/dL)	192.4 (43.0)	194.8 (36.5)	193.3 (23.4)	0.664
HDL Cholesterol (mg/dL)	44.5 (9.6)	42.1 (7.4)	40.6 (5.8)	0.786
Triglyceride (mg/dL)	133.9 (53.5)	134.3 (55.9)	135.8 (61.6)	0.452
Fasting glucose (mg/dL)	89.7 (11.9)	88.8 (12.1)	90.2 (15.2)	0.338
Creatinine (mg/dL)	0.9 (0.2)	0.9 (0.1)	0.9 (0.1)	0.778
RAP (mmHg)	7.5 (2.5)	9.1 (3.2)	11.3 (4.4)	<0.001
PASP (mmHg)	27.5 (5.3)	42.1 (2.9)	58.6 (10.2)	<0.001
TAPSE (mm)	21.8 (4.1)	19.3 (4.8)	19.0 (5.0)	0.016
EF (%)	52.7 (10.7)	46.2 (12.3)	45.2 (12.2)	<0.001
E/A ratio	1.0 (0.6)	0.7 (0.3)	0.3 (0.7)	<0.001
WMSI	1.5 (0.3)	1.7 (0.3)	1.9 (0.2)	<0.001
Dual antiplatelet therapy (%)	48.2	54.3	61.0	0.091
Statins (%)	39.1	49.0	53.0	0.111
Diuretics (%)	25.2	27.2	29.7	0.564
ACE inhibitor (%)	51.1	62.1	77.0	0.083
Beta-blocker (%)	45.6	45.2	55.1	0.221

Distribution of population by Pulmonar Hypertension probability. BMI: Body mass index; HT: Hypertension; CAD: Coronary artery disease; COPD: Chronic obstructive pulmonary disease; HR: Heart rate; RAP: Right atrial pressure; PASP: Pulmonary artery systolic pressure; TAPSE: Tricuspid annular plane systolic excursion; EF: Ejection fraction; WMSI: Wall motion score index. Data are presented as the mean and standard deviation (SD) for variables with normal distribution, as the median and SD for variables with not-normal distribution, and as frequency (percent), where appropriate. *p*-value: Significance level for ANOVA for continuous variables and chi square test for categorical variables.

**Table 3:** Predictors of PASP in the multivariate analysis.

	PASP in Overall Population		PASP in Intermediate and High Probability of PH	
	***β***	*p*	***β***	*p*
Age (year)	0.041	<0.001	0.022	0.451
Female sex (%)	−0.039	<0.001	−0.092	0.007
BMI (kg/m^2^)	0.021	0.667	0.013	0.312
BP (mmHg)	0.010	0.771	0.007	0.776
HR (bpm)	0.009	0.661	0.006	0.654
LA size ()	0.056	<0.001	0.018	0.711
LV size ()	0.007	0.887	0.002	0.878
RA size ()	0.095	<0.001	0.064	0.611
RV size ()	0.244	<0.001	0.265	<0.001
LV mass index (g/m^2^)	0.002	0.711	0.001	0.989
LVEF (%)	−0.050	0.001	−0.024	0.554
E/A ratio	0.008	0.443	0.004	0.565
TAPSE (mm)	−0.031	0.008	−0.025	0.445

PASP: Pulmonary artery systolic pressure; BMI: Body mass index; BP: Blood pressure; HR: Heart rate; LA: Left atrial; LV: Left ventricular; RA: Right atrial; RV: Right ventricular; TAPSE: Tricuspid annular plane systolic excursion; LVEF: Ejection fraction. *p*-value: Significance level for stepwise multivariate logistic regression analysis.

## Data Availability

The data underlying this article will be shared upon reasonable request from the corresponding author.
